# French recommendations for the management of glycogen storage disease type III

**DOI:** 10.1186/s40001-023-01212-5

**Published:** 2023-07-24

**Authors:** Camille Wicker, Aline Cano, Valérie Decostre, Roseline Froissart, François Maillot, Ariane Perry, François Petit, Catherine Voillot, Karim Wahbi, Joëlle Wenz, Pascal Laforêt, Philippe Labrune

**Affiliations:** 1 Maladies métaboliques et hépatiques pédiatriques, CHRU Hautepierre, 1 Avenue Molière, 67200 Strasbourg, France; 2 Centre de Référence des Maladies Héréditaires du Métabolisme- CHU La Timone Enfants, 264 rue Saint-Pierre, 13385 Marseille cedex 5, France; 3 Institut de myologie, Groupe Hospitalier Pitié-Salpêtrière, APHP. Université Paris Sorbonne, 47-83 boulevard de l’Hôpital, 75651 Paris Cedex 13, France; 4 Centre de Biologie et pathologie Est, maladies héréditaires du métabolisme, HFME, 59, Boulevard Pinel, 69677 Bron Cedex, France; 5 Médecine Interne, Centre Référence Maladies Métaboliques, hôpital Bretonneau, 2 boulevard Tonnelé, 37044 Tours cedex 9, France; 6 Pédiatrie, Centre de Référence Maladies Héréditaires du Métabolisme Hépatique, Hôpital Antoine Béclère, APHP Université Paris-Saclay, 92141 Clamart Cedex, France; 7 Laboratoire de génétique, Hôpital Antoine Béclère, APHP. Université Paris-Saclay, 92141 Clamart Cedex, France; 8 Service de cardiologie - Hôpital Cochin, APHP. Université Paris Centre, 27 rue du Faubourg Saint-Jacques, 75014 Paris, France; 9 Service d’hépatologie et transplantation hépatique pédiatriques, hôpital Bicêtre, APHP. Université Paris-Saclay, 94276 Le Kremlin Bicêtre Cedex, France; 10 Neurologie, Centre de Référence Maladies Neuromusculaires Nord/Est/Ile de France Hôpital Raymond Poincaré, AP-HP, Université Paris Saclay, 104 Boulevard Raymond Poincaré, 92380 Garches, France

## Abstract

The aim of the *Protocole National De Diagnostic et de Soins/*French National Protocol for Diagnosis and Healthcare (PNDS) is to provide advice for health professionals on the optimum care provision and pathway for patients with glycogen storage disease type III (GSD III).The protocol aims at providing tools that make the diagnosis, defining the severity and different damages of the disease by detailing tests and explorations required for monitoring and diagnosis, better understanding the different aspects of the treatment, defining the modalities and organisation of the monitoring. This is a practical tool, to which health care professionals can refer. PNDS cannot, however, predict all specific cases, comorbidities, therapeutic particularities or hospital care protocols, and does not seek to serve as a substitute for the individual responsibility of the physician in front of his/her patient.

## Introduction

Glycogen storage diseases (GSDs) are rare inherited diseases caused by an anomaly of glycogen metabolism, affecting its synthesis, degradation, use in glycolysis, as well as the lysosomal metabolism. They can be classified into three types, according to affected tissues: hepatic, muscular, and hepatic and muscular GSDs.

Debrancher enzyme deficiency, also known as Cori–Forbes disease or Glycogen Storage Disease type 3 (GSD III), is a disorder of glycogen metabolism of variable clinical severity, characterised by an intracellular accumulation of structurally abnormal glycogen. This leads to defective glucose release from glycogen in the liver and sometimes in the muscles, whereas gluconeogenesis is normal [1, 2]. The frequency of GSD III is 1 in 100 000 births, and approximatively 200 patients are living in France.

The first clinical manifestations appear in the first months of life with mainly metabolic signs, such as short fasting hypoglycaemia (less than 4 to 5 h) with no hyperlactataemia (lactates are low when fasting or hypoglycaemic as they are used in gluconeogenesis). On the other hand, there is post-prandial hyperlactataemia and a partial response to glucagon, contrary to what is generally observed in GSD I type I. Hepatomegaly and elevated transaminases may also be early manifestations.

The first signs of muscular impairment are often limited in childhood to an increase in CPK associated or not with muscular fatigability. Hypertrophic cardiomyopathy can occur in the first years of life. As years pass, hepatic symptomatology tends to resolve itself in favour of increasing muscular impairment, but hepatic fibrosis can occur, and, more rarely, cirrhosis and hepatocellular carcinoma. The muscular impairment can become invalidating to the point of requiring a wheelchair, and it can have consequences on the patients’ social and professional integration [3–6].

The treatment mainly relies on dietary measures, but there is, however, a greater fasting tolerance than in GSD type I. Apart from the carbohydrate supply, preserving gluconeogenesis requires an increased protein intake.

## Goals of the national diagnostic and care protocol

### Aims

The aim of the *Protocole National De Diagnostic et de Soins/*French National Protocol for Diagnosis and Healthcare (PNDS) is to provide advice for health professionals on both optimum care provision and pathway for GSD III patients.

This is a practical tool, to which physicians can refer when providing care for this disease, notably when establishing the care protocol in collaboration with both advising doctor and patient. PNDS cannot, however, predict all specific cases, comorbidities, therapeutic particularities or hospital care protocols, and does not seek to serve as a substitute for the individual responsibility of the doctor vis a vis his/her patient.

### Work method

This PNDS was drafted following the “Methodology for setting a national protocol for the diagnosis and care of rare diseases”, published by the *Haute Autorité de Santé*/French National Authority for Health in 2012 (methodological guide available on: http://www.has-sante.fr/). It is based on a critical analysis of the international literature.

The content of the PNDS was written and validated by a multidisciplinary working group, whose proposals were submitted to a separate group for rereading. Patients association has been involved in both writing and rereading processes. The corrected document was discussed and validated by a multidisciplinary expert group during ten telephone conferences.

### Links of interest

All participants in writing this PNDS filled out a public interest declaration.

The project is independent since there was no participation from anyone working in the pharmaceutical industry.

## Pathophysiology

**Fig. 1:**
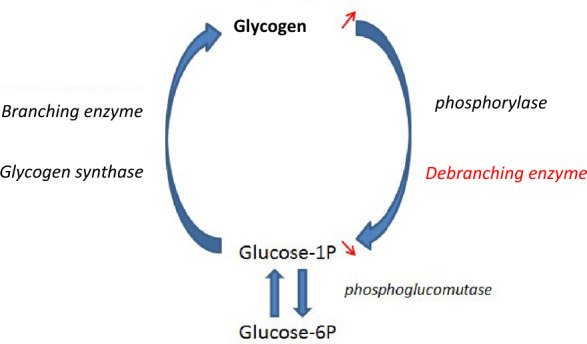
Simplified glycogen metabolism: synthesis and degradation cycle and consequences in cases of debranching enzyme deficiency

Glycogen is formed from glucose-1P by the action of two main enzymes (glycogen synthase and branching enzyme) making a branched chain polymer of glucose units. It constitutes the storage form of glucose for the cells. Two enzymes are involved in enabling glucose release from glycogen: the phosphorylase which enables the glucose 1-P units of the chains to detach and the debranching enzyme which enables the degradation at branch level (Fig. 1).

The debranching enzyme present in the liver and muscles is produced from the same gene, called *AGL* [7, 8]. When the debranching enzyme is deficient, glycogen is no longer completely degraded and accumulates in abnormal form with short secondary chains known as α-amylase resistant and limit dextrin.

Hypoglycaemic attacks are one of the first symptoms, but these are less severe than in GSD type I since some of the glucose of the glycogen can still be mobilised by the phosphorylase and because gluconeogenesis remains functional. The accumulation of abnormally structured glycogen in the liver is responsible for hepatomegaly and an increase in transaminases. There is also an increase in triglycerides but not in uric acid. During periods of fasting, the energy deficit leads to an increase in ketone bodies without increasing the lactate (since it is used in gluconeogenesis). Postprandially, the glucose absorbed is metabolised by alternative pathways such as through conversion to lactates, which could explain hyperlactataemia.

Regarding histopathology, muscle biopsies always show massive glycogen accumulation on PAS staining, with diffuse vacuolisation of the muscle fibres. These morphological anomalies are more marked than for other muscular GSDs, and are highly evocative of GSDIII. Muscle fibre necrosis and regeneration, and variable degree of fatty substitution are also observed [4].

Hepatic histological findings show a distension of hepatocytes linked to an overload of glycogen and periportal septal fibrosis. Other histological anomalies have been described: steatosis, hepatocellular ballooning and, more rarely, centrilobular fibrosis. Hepatic lesions can transform into adenomas or even hepatocellular carcinomas. There is no correlation between hepatic damages and muscular damages [3, 4].

Histological studies carried out on myocardiac biopsies show, in patients with left ventricular hypertrophy, glycogen accumulation in cardiomyocytes, with no architectural disorganisation of muscular fibres. This aspect is different from that observed in other hypertrophic cardiomyopathies, notably those of sarcomeric origin [4].

## Diagnosis and initial assessment

### Professionals involved

GSD III is most often diagnosed at paediatric age. Warning signs are thus generally identified by a paediatrician or a general practitioner. When the diagnosis is made in adulthood, warning signs may be identified by a general practitioner, neurologist, hepatologist, internist or endocrinologist.

Whichever practitioner suggests the diagnosis, it should be confirmed in an approved centre of reference, where therapeutic decisions should also be made.

References centres have been created by the French ministry of health in 2005. They must have a sufficiently large number of patients with a group of rare diseases, i.e. metabolic or neuromuscular, and gather experts in a same place. All these centres are organised in national networks (filières).

In these centres, the following professionals are involved in the diagnosis:

- Metabolic physician: for children it is a specialised paediatrician, and for adults it may be a specialised internist, or endocrinologist, or, more rarely in France, a specialised hepatologist
- Cardio-paediatrician or cardiologist
- Radiologist
- Geneticist and biochemist: diagnosis tests are only available in specialised laboratories.
- Physiotherapist
- Potentially neurologist, hepatologist.

### Clinical picture

#### Metabolic symptoms

There is a clinical variability in patients with GSD III: although the majority (85%) have hepatic and muscular damages, of variable degrees (GSD IIIa), 15% of the subjects present isolated hepatic damage (GSD IIIb) [1–3].

The most frequent manifestations at the time of diagnosis are hepatomegaly (98%), associated with hypoglycaemic episodes (53%), and failure to thrive (49%). The starting age is variable, with median ages of presentation ranging from a few months of life to 8 years [3].

The symptomatology of GSD III is generally less severe than in type I regarding the carbohydrate balance: fasting tolerance is variable but generally longer and hypoglycaemic episodes tend to be less severe. The early hepatomegaly sometimes stabilises later (generally at puberty) until it disappears completely on palpation in adulthood, probably in relation to an increase in fibrosis. Some complications such as cirrhosis, adenomas, and hepatocellular carcinomas have been described, justifying hepatic monitoring throughout the patient’s life [1–3].

#### Muscle symptoms

##### Skeletal muscle involvement

Skeletal muscle impairment often starts in childhood with abnormal fatigability during exercise, or proximal lower limbs weakness with Gower’s sign on physical examination. This muscular fatigability is sometimes associated with hypoglycaemic events that can also occur during exercise.

In adulthood, some patients develop permanent lower limbs muscle weakness with difficulties for climbing stairs, getting up from a chair, etc. In most cases, these symptoms remain moderate with slight progression; but some patients may require a stick for walking, and exceptionally, after the age of 40–50 years, may end up in a wheelchair. Distal muscle weakness is also frequent. Manual dexterity is often impaired at a young age, and can result in a lack of precision when writing. Grip strength is decreased. Hand atrophy can also be observed in severe cases. Tibialis anterior muscle weakness can also be observed early, with difficulties for walking on the heels [3, 4].

Patients more rarely complain of significant pain during exertion, and never present acute rhabdomyolysis episodes (CK levels are permanently moderately increased), in contrast with McArdle's disease (GSD type V), the most frequent muscle GSD which is characterised by muscle pain and exercise-induced rhabdomyolysis attacks.

Respiratory insufficiency remains exceptional [3, 4], as respiratory muscles are rarely involved.

##### Cardiac involvement

The majority of patients have no heart symptoms. In the presence of hypertrophic cardiomyopathy, symptoms of cardiac failure can exist in both adults and children [4]. This is primarily on the left ventricle and characterised by dyspnoea and functional limitation on exertion. These symptoms are mainly linked to diastolic cardiac dysfunction with, in some cases, a component linked to a left intraventricular obstruction. More rarely, in the most developed forms of cardiomyopathy, dyspnoea can be connected to an altered systolic function. Less frequently, some patients can also present palpitations, faintness and precordial catch syndrome linked to functional myocardial ischaemia. The occurrence of sudden cardiac deaths has been reported in the most severe forms of cardiomyopathy, whatever the patient’s age: the suspected mechanism is the occurrence of ventricular rhythm disorders on a fibrotic myocardium [3, 4].

### Confirmation of the diagnosis

#### Biochemistry

The diagnosis of GSD III is initially suspected in response to an association of clinical symptoms and simple biochemical results that can easily be achieved in routine practice (see non-specific biochemical tests).

Biological confirmation of the diagnosis then relies on two complementary approaches that can be carried out sequentially, concomitantly or separately [9, 10].

The first approach is biochemical and relies on measuring the activity of the debranching enzyme (amylo-1,6-glucosidase) most often in leucocytes, potentially associated with a measurement of the glycogen content in red blood cells (see biochemical diagnosis).

The second approach is molecular and can be carried out at the start (following functional explorations or when there is a family history) or to confirm an abnormal biochemical work-up. In some countries, genetic testing is the preferred and only method.

##### Non-specific biochemical tests

Relatively short fasting hypoglycaemic episodes can occur, associated with ketosis, post-prandial hyperlactacidaemia, hypertriglyceridaemia and an increase in transaminases which are generally relatively high during the first decade of life and decrease later on (note that the high levels of transaminases and particularly ASATs can be of muscular origin). Lactataemia evolves following a curve parallel to that of glycaemia, with a trend towards post-prandial hyperlactacidaemia and fasting hypolactacidaemia (due to a preservation of gluconeogenesis).

CK levels are frequently elevated, sometimes in patients without muscle symptoms, and CK level should be assessed systematically. Uric acid serum concentration is generally normal [10].

##### Specific biochemical tests: enzyme activity

These highly specialised analyses can only be done in very few laboratories in France [9, 10]. They are most often carried out on blood samples after isolating erythrocytes and leucocytes, but they can also be used when examining a muscular biopsy (which is however not indispensable for the diagnosis).

Hepatic biopsies are no longer used for biochemical diagnosis.

- Glycogen measurement [10].

A sharp increase in glycogen content measured in red blood cells occurs when the patient is fasting.

The increase is more variable in the muscles.

- Measurement of debranching enzyme activity [10]

The diagnosis relies on evidencing a deficient activity for this enzyme, measured most often in total leucocytes. Exceptionally, it can be measured in cultured fibroblasts or muscle biopsy.

#### Genetic diagnosis

The *AGL* gene comprises 35 exons of which 33 are coding (start codon in exon 3) and two exons 1 and 2 which are alternative noncoding (hepatic and muscular). It is located on the short arm of chromosome 1 at 11p21.2. Six isoforms have been described and the major isoform codes for a protein made up of 1532 amino acids.

The majority of the 210 variants classified as pathogenic or probably pathogenic reported in the ClinVar database are nonsense mutations or variants leading to a reading frame shift with the appearance of a premature stop codon (deletion, insertion, duplication out of frame or mutation in the splice sites), responsible for the absence of residual enzyme activity [11–15]. Some variants characteristic of ethno-geographic groups, such as the deletion c.4556delT in Sephardic Jews, have been reported. In France, variants other than missense ones represent more than 93% of the harmful recessive genes identified. The deletion c.3216_3217delGA and the nonsense mutations c.3980G > A (p.(Thr1327*)) and c.256C > T (p.(Gln86*)) alone represent a quarter of all of the harmful recessive genes identified [11–15]. The penetrance is considered to be complete. The genotype–phenotype relationships are difficult to establish, apart from a reported link between the presence of mutations in exon 2 (i.e. c.17_18delAG) and the purely hepatic form [11–13].

The genetic diagnosis is traditionally carried out by sequencing the coding regions of the *AGL* gene. A study of this gene is carried out when clinical–biochemical indications are strong. But a gene panel analysis including the *AGL* gene by high-throughput sequencing (NGS) can lead to GSD III diagnosis in cases of isolated muscular symptomatology in adulthood (rare form) and when there are few indications or when they lack specificity [11–15]. This is the case in particular of gene panels involved in hepatic pathologies, hypoglycaemic episodes, GSDs or myopathies. For adult forms, the “filière FILNEMUS” (national network for neuromuscular diseases) working group proposed establishing a list of genes in the form of analysis panels for myopathies. They classified the *AGL* gene in the list of priority genes to be sequenced in cases of suspected metabolic myopathy [14].

#### Abdominal ultrasound

Abdominal ultrasound allows measuring liver size and is often part of establishing the definite diagnosis. Hyperechogenic hepatomegaly is often present. This will then be repeated during the monitoring, notably to look for hepatocellular adenomas or signs of fibrosis or liver cirrhosis.

### Initial assessment

#### Cardiological work-up

Prevalence of left ventricular hypertrophy is high. It ranges from 27 to 86%, depending on the characteristics of the different cohorts published, notably the average age of patients included and the criteria used for diagnosing hypertrophy. The pattern of this hypertrophy is generally symmetrical, meaning that the thickening of the left ventricular walls takes place homogenously across the different walls. The main aim of the cardiological work-up is to screen for hypertrophic cardiomyopathy and to guide the treatment regimen, notably in the more severe forms associated with cardiac symptomatology, for which conventional cardiological treatments or dietary modifications can be indicated.

The risk of conduction disorders and supraventricular or ventricular rhythm disorders appears lower in comparison to the risk of hypertrophic cardiomyopathies of sarcomeric origin. The risk of arrhythmia could be underestimated, notably because of the low prevalence of these pathologies. Rare cases of sudden death have been reported in children and adults [3, 4].

##### Electrocardiogram

An abnormal electrocardiogram was reported in more than 80% of GSD III children and adults the most frequent criteria of left ventricular hypertrophy are tall R waves in the right precordial leads and Q waves in the lateral inferior leads, and precordial repolarisation abnormalities, most often with asymmetric negative T waves and sometimes a ST segment sub-shift. It is unusual to observe conduction disorders or a pre-excitation syndrome, in contrast to other cardiac GSDs [16].

##### Echocardiography

Echocardiography is a first-line test used to screen for and assess hypertrophic cardiomyopathies [4]. It is necessary when establishing a diagnosis, and should be repeated during the follow-up. It enables a diagnosis of left ventricular hypertrophy which is normally symmetric (relationship between the septal and posterior thicknesses < 1.3) following the usual measurement criteria for parietal thickness and to a lesser extent the mass [17, 22]. It also enables an assessment of (1) the left ventricular ejection fraction (rarely reduced); (2) the diastolic function parameters, often abnormal, sometimes with an elevated left ventricular filling pressure; (3) the presence of a left ventricular obstruction, most often mid-ventricular, rarely subaortic; (4) the presence of an apical aneurysm, exceptionally reported in forms with mid-ventricular obstruction; (5) a right ventricular hypertrophy that may exist in the most severe forms [4, 23–26].

##### 24-h Holter monitoring

This test aims at refining the diagnosis of cardiac rhythm disorder. It seems reasonable to offer this to patients with hypertrophic cardiomyopathy (every 1–2 years depending on the severity) or in the presence of palpitations, faintness or syncopes [16–18].

##### Cardiac MRI

Cardiac MRI is useful to obtain a finely detailed characterisation of the myocardial damage. Apart from providing a better appreciation of the anatomy and notably of the degree of hypertrophy than ultrasound, this technique also enables the tissues to be characterised, and is particularly useful searching for myocardial fibrosis through the presence of delayed enhancement (fibrous scars) or quantitatively on T1 sequences (T1 mapping, extracellular volume/ecv measurements). The implications of MRI data for care provision have not been established for GSD III, but the presence of myocardial fibrosis could encourage more frequent cardiac monitoring and more intensive therapeutic care [3, 4, 19–23].

MRI could be discussed when the ultrasound identifies ventricular hypertrophy, in children old enough for not being obliged to do the MRI under general anaesthesia (meaning most often over 7 years), or in adults. This test could be repeated every 5 years (owing to the complexity of diagnostic efforts).

##### Blood assays of cardiac natriuretic peptides: BNP and NT-proBNP

These biomarkers are used routinely for both diagnosis and monitoring of systolic and diastolic heart failure. They can also be used in GSD III patients presenting left ventricular hypertrophy, even asymptomatic, since they can make it possible to identify heart failure, notably diastolic, at an early stage, and then monitor the evolution over time. An assay at pre-symptomatic stage could serve as a reference later on, if symptoms appear that raise the suspicion of heart failure. In patients presenting exercise-related dyspnoea, a high level of these biomarkers could help obtain a positive diagnosis. These assays can easily be incorporated into the annual biological work-up programmed in the context of general follow-up for the pathology. However, to date, no reported data support their use in routine practice [3, 4].

#### Muscle assessments

Skeletal muscles evaluation most often show mild impairment in younger patients. With age, the muscle impairment can worsen, often after 30 years of age [27–29]. In rare cases, the severity of lower limbs muscle weakness may require wheelchair assistance, generally after the age of 40 years. The initial work-up should be performed in a neuromuscular reference centre, by trained neurologists and physiotherapists. It will then serve as a reference for the subsequent follow-up [31–35].

An occupational therapy assessment is proposed annually. Orthopaedic care could also be needed.

#### Posture and range of motions

Alignment verification: spinal, pelvic, genu valgum/recurvatum, ankle and foot.

The base of support in standing position is often wide.

Range of motions: frequent hyperextension of elbows and knees.

The most common posture defect, characterised by an anterior pelvic tilt and wide base of support, can be the result of the hepatomegaly.

##### Function tests and muscle strength measurement [31, 32]

Motor Function Measure (MFM) scale: evaluation of gross motor skills. (https://mfm-nmd.org/).

Brooke and Vignos scales: situate the functional capacities of the limbs [30].

6-minute walk test: evaluation of muscular, and cardio-vascular capacities [33].

Timed tests such as getting up from a chair, climb 4 stairs as quickly as possible, etc.

Assessment of manual dexterity using the Purdue pegboard [35].

These tests are not used in children, but are regularly performed in patients 14 years of age and older.

#### Other tests

##### Muscle MRI

Muscle MRI is now largely used in neuromuscular centres, allowing assessment and quantification of the severity of muscle involvement and fatty replacement of skeletal muscles. It is a non-invasive tool, and in our experience should be performed preferentially in adults because muscle lesions generally occur at adult age in GSD III. Therefore, this exam is probably much less important for the follow-up during childhood [36, 37].

The main limitations of this exam are claustrophobia and severe respiratory insufficiency which prevent patients from lying down, but this complication is exceptional.

##### ENMG (electroneuromyography)

Electroneuromyography is not systematic and most often detects non-specific myopathic features during detection. It should, however, be systematically performed when patients complain of sensory symptoms, or when characteristics and progression of muscle weakness are unusual and severe.

The possible coexistence of peripheral nerves involvement remains debated, and is suggested in adult patients presenting distal muscular weakness [38–41]. This distal muscular weakness is probably the consequence of distal myopathy, and a recent study of a series of 16 patients, who all underwent an ENMG following the standard protocol, did not show any sign of peripheral nerve damage [42].

##### Exercise tests

Two different exercise tests can be performed requiring specific equipment with increased monitoring of the risk of hypoglycaemia: the forearm effort test or “grip-test”, and the exercise test on an ergocycle or treadmill. These tests should only be performed in reference centres on a case-by-case basis. Their main aim is to document and characterise the patients’ muscle metabolism impairment during exercise [31, 32].

##### Muscle biopsy

Muscle biopsy should not be used for diagnostic purposes since the diagnosis is made in the vast majority of cases in childhood via enzymatic assays and/or genetic tests. They can, however, lead to the diagnosis in rare cases in adults, when muscle symptoms are predominant, with moderate hepatic manifestations in childhood that were not recognised as GSD.

When performed, muscle biopsy always shows massive glycogen accumulation after PAS staining, with large vacuoles of muscle fibres [43]. Focal endomysial fibrosis with chronic inflammation may also be found. These histopathological abnomalies are more marked than in other muscle GSDs, and may suggest GSD III [43–45].

##### Hepatic imaging

###### Hepatic MRI

As a complement to abdominal ultrasound, and especially when hepatic lesions have been detected, abdominal MRI with injection of a contrast medium is indicated for the monitoring and precise diagnosis of these lesions. It is ideally performed annually from the age of 10 years (age at which the children are able to undergo this test without moving), or more frequently in cases of potentially progressive lesions. It also makes it possible to assess the indirect signs of cirrhosis [46–48]. MR elastography performs better than MRI for the detection of fibrosis, but no data are available to date in GSD III.

###### Fibroscan^R^—hepatic elastography

Although this technique is frequently indicated to assess hepatic fibrosis in many chronic liver diseases, its use in GSD III has yet to be validated. In children, if the technique is available in an experienced centre, using it for a longitudinal monitoring to screen for the appearance of fibrosis can be beneficial, whatever the patient’s age. Patients then act as their own control, throughout the monitoring.

##### Psychological assessment

The neuropsychological aspect of the disease is rarely addressed in the different recommendations or studies of cohorts of patients with GSD III. Clinicians nevertheless recognise that this disease frequently has consequences on both patients’ and patients’ families psychological and emotional wellbeing [3–5]. Psychological support may prove necessary.

##### Neuropsychological assessment and brain MRI

Consequences of repeated hypoglycaemic episodes on psychomotor development remain unknown since little data are available. In the ISGSDIII cohort, the vast majority of patients were considered as intellectually normal, and only 3% of the patients were considered to have a ‘low’ or ‘limited’ cognitive ability [3, 4]. A study of patients’ cognitive profiles showed deficits in social cognition and executive function, with no anomalies on brain MRI [5]. The problems observed could explain some of the economic and social difficulties experienced by the patients, and the difficulties they have in observing regular medical follow-up. In practice, as for other metabolic diseases, brain MRIs and neuropsychological tests are not indicated systematically [5] but can be discussed on a case-by-case basis depending on the clinic. Some patients may benefit from social support.

##### Screening for orality disturbances

Like in all chronic diseases involving a strict diet, with frequent meals at fixed times and sometimes nocturnal enteral feeding, orality disturbances should be screened for systematically at every consultation, especially in children (patients who are unable to brush their teeth, are only accepting mashed food and soups, cannot chew and find it hard to swallow small bits of food).

### Differential diagnoses

The differential diagnoses for GSD III depend on the age of the patient. During childhood when hepatic symptomatology is predominant, main differential diagnoses are other hepatic GSDs:

- GSD type I

It manifests itself by hypoglycaemic episodes (generally more severe) and hepatomegaly. Glycaemia and lactataemia progress by cross-reacting with hyperlactacidaemia during hypoglycaemic periods, and no ketosis. CPKs are most often normal. There is hypertriglyceridaemia and hypercholesterolaemia, as well as hyperuricaemia. Transaminases may be normal or moderately increased. Neutropenia or even inflammatory damage to the digestive tract can be present in type Ib. There are no signs of muscular impairment.

- GSD type VI

Liver phosphorylase deficiency leads to hepatic damage that is generally more moderate with non-severe or even absent hypoglycaemia. Transaminases are moderately increased and CPKs are normal. The hepatomegaly present in childhood tends to disappear with puberty. There are neither signs of muscular nor cardiac damage.

- GSD type IX

The deficiency of phosphorylase kinase in the liver can be linked either to a deficiency of a purely hepatic subunit (subunits A2 and G2) or to a deficiency of a subunit shared with the muscular enzyme (subunit B). Hypoglycaemia and hyperlipidaemia are variable and generally moderate. They attenuate in adulthood. Hepatomegaly is present early in childhood and is associated with failure to thrive. There is an increased risk of hepatic fibrosis. In the subunit B deficiency, discrete muscular hypotonia is also noted. The transmission is X-linked for the subunit A2 deficiency and autosomal recessive in deficiencies of subunits G2 and B.

If the disease was not diagnosed in childhood, and since hepatic symptomatology becomes more discrete and muscular impairment more important with time, the main differential diagnoses in this case are other muscular GSDs, as well as other metabolic myopathies and muscular dystrophies.

### Possible complications

#### Hepatic

The liver is one of the target organs of GSD III, but hepatomegaly and fasting tolerance generally improve with age.

However, since life expectancy of GSD III patients is lengthening, we are beginning to better understand the long-term complications of the disease and there are perhaps more to discover. Several publications have demonstrated a cirrhogenic evolution. A retrospective study published in 2017 assessed the risk of severe liver damage in adulthood to be about 15% of the population [3]. Another more recent study, on 25 older patients with GSD III, has reported liver cirrhosis in about 40% of patients [48]. Cirrhosis can progress towards end-stage liver failure [4]. Some articles have also reported the occurrence of hepatic adenomas in patients living with GSD III. However, these adenomas occur much less frequently than in the course of GSD type 1 [3, 48].

Finally, observations have also been published of hepatocellular carcinomas in patients with GSD III, generally following cirrhogenic evolution [46–48]. To date, there are no reliable biomarkers to confirm that cirrhosis has evolved to hepatocellular carcinoma. Alpha-foetoprotein should be measured regularly, but the results should be interpreted with caution and normal values should not rule out hepatic imaging.

The main biochemical abnormality present over the years is high serum transaminase concentrations. There is no manifestation of liver failure, excepting in the final stages of decompensated cirrhosis. Biochemical monitoring relies on measuring prothrombin time, ASAT, ALAT, albumin and bilirubin. In adults, it is important to ensure that the high levels of transaminases are not due to another cause (chronic liver disease, virus B or virus C infection, medicinal toxicity, autoimmune disease, NASH).

In young children, hepatic imaging monitoring involves annual ultrasounds. In older patients, starting in the second decade of life, this is completed with an annual hepatic MRI with injection of a contrast agent, since this enables an early detection of intrahepatic nodules, and evidences both direct or indirect signs of the development of cirrhosis, adenoma or hepatocellular carcinoma [3, 46–48].

Hepatic elastometry techniques such as Fibroscans^R^ can help to monitor the appearance of fibrosis, although they have not been validated for this pathology.

#### Endocrinological and nutritional

Patients generally present growth retardation in childhood, but the majority then catch up to reach normal height by adulthood.

Additionally, about 25% of the patients, like in type 2 diabetes, are overweight in adulthood.

Finally, female patients often present polycystic ovary syndrome; however, no effects on fertility in adulthood have been evidenced [49].

The pathophysiology of these endocrine manifestations is not yet well understood, but insulin resistance (due to regular ingestion of glucose and excess body weight) appears to be one of the main factors [50, 51].

#### Bones

Bone complications are relatively frequent in patients with GSD III. Osteopenia or even osteoporosis can result from the addition of several factors: myopathy, metabolic imbalance (notably hypercholesterolaemia and hypertriglyceridaemia), and occasionally chronic ketosis in cases of recurrent hypoglycaemia [52].

Bone monitoring should be done regularly by osteodensitometry, at a frequency that depends on the severity of bone damage [52]. Vitamin D supplements are required. Additionally, muscular reeducation can contribute to improve bone trophicity.

### Announcing the diagnosis and genetic advice

The diagnosis should be announced during a dedicated consultation. It should be done by a physician who knows the disease well, and if possible, in the presence of both parents. It includes an explanation of the diagnosis and the complications, planning of monitoring and therapeutic possibilities, genetic advice (screening siblings), and a request for consent to genotyping. It is particularly important since it is the basis of the quality of subsequent follow-up, and makes it possible to put in place essential support for the child and his/her family [3, 4].

This is an autosomal recessive inherited pathology. Genetic advice should be offered to couples when genetic results are available. Both parents are generally heterozygous carriers of one of the two mutations found in their child. There is a 25% risk of recurrence for each pregnancy.

If the pathogenic variants have been identified and characterised in the parents, prenatal diagnosis is possible on chorionic villi sampling or amniotic fluid [3, 9, 10].

Preimplantatory genetic diagnosis may potentially be proposed. However, since GSD III is a disease of both variable severity, and intra-familial phenotypic expression, prenatal diagnosis should be discussed on a case-by-case basis and its indication should be validated in the context of multidisciplinary prenatal diagnosis meetings within the CPDPN *(Centre Pluridisciplinaire de Diagnostic Prénatal /* Multidisciplinary Centre for Prenatal Diagnosis).

### Therapeutic care provision

#### Objectives

- Instigate a specialised dietary treatment to prevent hypoglycaemic episodes and ensure optimal metabolic balance that allows satisfactory growth.
- Take a therapeutic education approach to enable the patient to become progressively independent in managing his/her disease and long-term treatment, and to act appropriately in emergency situations.
- Treat any potential cardiac failure or rhythm disorder and limit the progression of the cardiomyopathy if present.
- Instigate functional muscular care package to limit the impact of muscle impairment on patients’ daily lives.
- Provide support during pregnancy for affected women.
- Manage any potential complications.
- Consider non-specific complementary care if required (psychological support, social worker, etc.).

#### Metabolic care: diet

##### Principle of the dietary treatment

The aim of the dietary treatment in childhood is to prevent hypoglycaemic episodes and ensure a metabolic balance that enables satisfactory growth [3, 6, 53, 54].

For this, blood glucose levels should always be above 0.5 g/L (2.8 mmol/L). For an optimum metabolic balance, they should remain between 0.60 g/L (3.3 mmol/L) and 1.2 g/L (6.6 mmol/L).

The dietary treatment prescription should be adapted to clinical signs (particularly in cases of cardiac failure) and to biochemical work-up (blood glucose cycle, hepatic work-up, lipid profile, etc.) [56].

This specific feeding regime should be individualised for each patient, despite the generalities in the dietary treatment [1–3].

To reach these objectives, the diet in young childhood tends to be high in carbohydrates, with intake of bot raw and cooked starches [57, 58].

Food intake is divided into portions to be taken at set times according to fasting tolerance, during the diurnal cycle. The family’s lifestyle is also taken into account:

- Daytime: 6–4 regular food intakes, every 3–4 h.
- Nighttime: introduction of continuous enteral feeding (CEF) if fasting is badly tolerated, or of one to two snacks.

###### The dietary treatment should take into account age-related nutritional requirements, and be personalised

Specific nutrient balance from 0 to 18 years:

**Table:** 

Patient age	0–3 years	3–10 years	10–18 years
Energy	Normal calorie supply for age The level of physical activity is taken into account		
Proteins	10–15% of TEI	15–20% of TEI	20–25% of TEI (Or even 30%)
Lipids	30–35% of TEI	30–35% of TEI	30% of TEI
Carbohydrates	55–58% of TEI	50–55% of TEI	45–50% of TEI (Or even 45–40%)
Micronutrients	100% of NRP Vit D supplements	100% of NRP Vit D supplements	100% of NRP Vit D supplements

*TEI* daily total energy intake determined by the NRP: ANSES notification 12/2016 Referral n°2012-SA-0103 *NRP* Nutritional Reference for the Population

The nutrient balance differs from recommendations for the general population. It is adapted to age categories and natural history of GSD III: a tendency towards high-carbohydrate intake in young childhood to prevent hypoglycaemia, with a progressive switch towards high-protein intake during childhood [59, 60]. Protein intake is increased as carbohydrates is decreased, to favour gluconeogenesis and limit glycogen storage in both liver and muscles.

##### Adapting the choice of foods

###### Whatever the patient’s age

Each meal should include foods rich in complex carbohydrates (during childhood) and in proteins of “high biological value” (meat, fish, eggs and all dairy products), without forgetting fruit and vegetables for their high fibre and vitamin contents [55].

Conversely to GSD I patients, there is no benefit to limit lactose and fructose.

On the other hand, the diet should be poor in sucrose (sugar) to limit the energy intake and avoid hyperglycaemic spikes.

The dietary regime, although specific, should also respect social, cultural and religious habits of the family. Despite the constraints of the adapted diet, unprocessed foods should be prioritised.

###### For infants

Breastfeeding on demand is possible up to 6 months (or even longer) but at a minimum of 8 feeds per day in the first months. This rhythm should be tailored to fasting tolerance (blood glucose cycle). Otherwise, all infant formula milks are suitable, stage 1, then stage 2.

All infant milks (breastmilk or formula) mostly contain lactose (disaccharide very rapidly hydrolysed and digested); this is why it is important to complement bottles or breastfeeds with maltodextrin (polysaccharide, slower to digest), to extend the fasting time.

During the second year of life, infant milks should be replaced with cow’s milk (semi-skimmed or whole) to increase the protein intake, while ensuring that recommended intakes of essential fatty acids (omega 3, ARA/DHA), vitamins and iron are covered.

###### Food diversification

This can start, like for all babies, around 4-5 months of age:

- Addition of sugar-free infant cereals (can replace maltodextrin).
- Introduction of vegetable mashes (half potato and half vegetable)
- Addition of a high-protein food (MFE: 10–20 g /mash) and vegetable fat (rapeseed, olive, sunflower, etc.)
- Introduction of sugar-free unflavoured dairy products and sugar-free fruit purees, alternated for dessert.

The diversification should continue throughout the 1st year (selection, texture, etc.) like for all young children, with a specific adaptation:

- Introduction of raw cornstarch around one year of age [57].
- Increase in the proportion of proteins in the ration (stimulates gluconeogenesis pathway from the first year onwards).

##### Nutrition during nightly fasting

There are two possibilities to reduce the nightly fasting time: dividing up food into several snacks or continuous enteral feeding (CEF).

###### CEF

When fasting tolerance is very short (< 6 h), notably in young children or adults in specific situations (pregnancy), it may be necessary to use CEF (nasogastric tube or gastrostomy).

To find the best solution, the decision should be made collectively, involving both multidisciplinary team and family [55].

Benefit–risk balance of CEF:

- During the period when nocturnal fasting time is short, a continuous supply of glucose makes it possible to obtain satisfactory growth thanks to better metabolic balance, and to avoid nocturnal awakening (child and families).
- The risk remains of severe hypoglycaemia in case of sudden stop of the enteral feeding pump (reactive hypoglycaemia by hyperinsulinism), and also during the programmed halt in the morning. Breakfast should thus be given within a maximum of 30 min after stopping the pump.
- The risk of aspiration is very low.

Carbohydrate requirements provided by CEF

**Table:** 

Age of the patients	Glucose supply in mg/kg/min
Infant to 6 years	8–5
School-age children	5–3
Adults (if CEF is indicated and/or necessary)	3–2

The first year of life, CEF is made of infant milk enriched with maltodextrin, to cover glucose requirements.

Around 1 year of age, ready-to-use nutritional paediatric mixes (pouches: homogenous, stable mix, hygienic, practical, etc.) can be prescribed.

Very often, nocturnal drip feeding is no longer necessary when children start primary school. This is replaced by two snacks during the night (once fasting time is greater than 6 h).

###### Night snacks

Before 1 year of age, night snacks made of milk and maltodextrin (± sugar-free cereals).

After 1 year of age, raw cornstarch progressively replaces maltodextrin in the milk (quantities adapted to both fasting time and digestive tolerance)

The benefit of snacks is that there is no risk of severe reactive hypoglycaemia.

##### Introduction of raw cornstarch

Raw cornstarch is introduced in the ration around 10–12 months of age [57–59].

This makes it possible to extend fasting tolerance.

The absorption of this very complex carbohydrate is delayed and spread out over time, which ensures glucose supply between mealtimes, thus making it possible to space out meals.

To amplify its delaying effect, cornstarch should be taken around 20–30 min after the end of either meals or snacks.

The introduction should be progressive and adapted to each child, to avoid causing digestive problems: gas, bloating, diarrhoea (linked to digestive immaturity).

The first corn starch proposed should be Maïzena®. After 2 years of age, it is then possible to use Glycosade® (Vitaflo laboratory, Nestle Health Science) as an alternative [57, 58]. This latter starch is modified, thus providing some patients with better metabolic control, by extending fasting time, compared to Maïzena®. It also improves digestive tolerance, at equal quantities. This type of starch is introduced during a blood glucose cycle work-up and metabolic assessment, to assess its efficacy [58].

###### What quantities and how to divide it up?

Quantities are adapted to each child, according to his/her weight and fasting tolerance. In this way, the daily division of tolerated and necessary raw starch doses is defined and prescribed [55, 57].

The aim is to obtain a significant increase in fasting tolerance**,** with no digestive troubles and without changing the child’s appetite:

- Daytime: 0.5 g–1 g of raw cornstarch per kilo of weight per intake
- Nighttime: 1 g–1.5 g of raw cornstarch per kilo of weight per intake.

The cornstarch is given raw, diluted in a cold liquid of twice the volume of the weight of the starch, and given alone or after food intake. To increase protein supply, the starch can be mixed with cold milk.

The rhythm of raw starch snacks is tailored for each child, and reviewed at every consultation (growth, school, sport, etc.).

Starting in adolescence, the quantities of raw starch should decrease progressively, to finally be interrupted in adulthood (except in specific cases).

##### High-protein food

###### The benefit of proteins in the dietary treatment

A higher protein intake compared to a normal diet will enable the activation of gluconeogenesis from glucogenic amino acids.

As soon as possible in adolescence, the protein intake should be increased, and carbohydrates decreased [60, 61].

The increase in the proportion of proteins or lipids should not result in an increase in overall energy intake. In order to follow recommendations, ensure that the proportion of carbohydrates is reduced [60, 61].

###### In practice, how to increase the protein content of food for young children?

- Increase the intake of meat, fish, eggs (with 2 main meals) and dairy products.
- Replace infant milk with cow’s milk during the second year of life.
- Possibly introduce protein powders (≥ 85% protein/100 g) during childhood.

Pay attention to the risks of high-protein diets: renal damage through high protein intake and increase in uric acid in particular, to be closely monitored.

##### Adult feeding

Nutrient balance in adulthood

**Table:** 

	After 18 years of age	Normal calorie levels adapted to the level of physical activity and life situation
Proteins	30% of TEI	High protein
Lipids	35% of TEI	Normal lipid to low lipid
Carbohydrates	35% of TEI	Low-carbohydrate
Micronutrients	100% of NRP Vit D supplements	

*TEI* daily total energy intake determined by the NRP: ANSES notification 12/2016 Referral n°2012-SA-0103 *NRP* Nutritional Reference for the Population

Like in children, “standard” nutritional balance is highly modified, focus should be on sharply increasing protein intake (checking that the patient has normal renal function) [60, 61].

Carbohydrate intake should be decreased in kind, especially of simple carbohydrates (saccharose, fructose).

Raw starch intake at night should be stopped progressively**,** (if this has not been done in adolescence) to be replaced by protein powders or high-protein food.

##### High-protein, low-carbohydrate, normal-calorie diet for patients with cardiomyopathy

In cases of severe cardiac damage, recommended diet is high in proteins, low in carbohydrates and high in lipids to complete the energy intake [62–65].

The aim of this diet is to reduce the risk of cardiac and muscular glycogen overload, while maintaining blood sugar levels by favouring gluconeogenesis from lactates and ketone bodies.

In GSD III, an overly high intake of carbohydrates can worsen cardiac damage, whereas cardiomyopathy appears to be reversible with the introduction of this specific nutritional treatment [64–66]. Some teams also noted an improvement in musculoskeletal damage after introducing this diet.

However, it is more burdensome than a usual diet. Thus, despite its efficacy, adhesion to the diet and impact on both family and social life should be assessed at each consultation.

Summary table of the division of nutrients for a normal energy intake in function to age:

**Table:** 

Age of the patients	0–3 years	3–10 years	> 10 years
Proteins %	12–15 of TEI	15–20 of TEI	20–25 of TEI
Lipids %	55–65 of TEI	55–65 of TEI	55–60 of TEI
Carbohydrates %	20–30 of TEI	20–25 of TEI	20–25 of TEI
Glucose infusion rate (mg/kg/min)	1–2	1	1
Late snack	Possible to replace night EF depending on the nocturnal fasting tolerance	Progressive introduction as enteral feeding stops	Systematic
Micronutrients	100% of NRP	100% of NRP	100% of NRP

*TEI* daily total energy intake determined by the NRP: ANSES notification 12/2016 Referral n°2012-SA-0103 *NRP* Nutritional Reference for the Population

This type of feeding varies depending on child’s preferences, appetite and compliance.

Until the age of 5 or even 8 years, nocturnal enteral feeding is often necessary with a very low carbohydrate intake: 1–2 mg/kg/min to maintain blood glucose levels > 3 mmol/L.

However, nocturnal enteral feeding is not systematic in childhood, particularly if blood glucose levels are high enough after a night of fasting. It is then necessary to introduce a snack at the beginning of the night, most often between 10 pm and midnight. This should be made of 1 to 2 g/kg of raw Maïzena® (depending on the child’s fasting tolerance, this is the only situation where cornstarch is prescribed in this type of diet), complemented with protein powder and lipids. The fasting tolerance should be checked during a hospital stay in order to set evening snack and breakfast times.

This type of feeding is prescribed alongside an addition of ketone bodies (3 OH sodium butyrate in 4–5 intakes per day) as an energy substrate for cardiac and skeletal muscles [67]. It is not necessary to prescribe a low-salt diet since ketone bodies do not contain sodium chloride but sodium hydroxybutyrate. In all cases, this very specific diet should be prescribed, undertaken and adapted in a specialised centre.

##### Specialised dietary follow-up and monitoring

Whatever the age of the patient, this requires a healthcare structure with a specialised metabolic dietician:

- Monitor growth, height and weight, if growth is not satisfactory (BMI chart) until target height is reached.
- In adults, monitor weight chart to maintain normal BMI.
- Regularly perform adapted biochemical metabolic work-ups with installation of a continuous glucose monitor (monitors blood glucose levels), to adapt rhythm and composition of food intakes and enteral feeding [68]. An alternative could be to prescribe a blood glucose meter and provide education for performing regular capillary blood tests at home.
- Realise a precise nutritional assessment with a food diary (monitoring the ingesta)

##### Resugaring

Hypoglycaemia is a fall in blood glucose levels to below the age-adjusted norms:

- Blood glucose below 0.47 g/L (2.6 mmol/L) in newborns and infants
- Blood glucose below 0.54 g/L (3.0 mmol/L) in older children
- Blood glucose below 0.60 g/L (3.3 mmol/L) in adults.

More individually, it could be defined as the blood glucose level at which neurological signs appear. This level is specific to each patient due to the individual capacity to use alternative energy substrates (ketone bodies, lactate).

When blood glucose level is below 0.50 g/L or 2.8 mmol/L:

- Keep the patient at rest.
- Give a quantity of sugar or glucose adapted to the patient’s weight:
  o 1 vial of 10 ml of G 30 (30% glucose) (or 3 g of glucose) or 50 to 100 ml of fruit juice for children (10 to 20 kg).
  p 5g of sugar for 20 kg of body weight (adolescent, adult).
- If hypoglycaemia occurs outside mealtimes, it is possible to then give a snack made of a food rich in complex carbohydrates (bread, biscuit, cereals) or raw starch (childhood) or a high-protein food (from adolescence onwards): milk, dairy products.
- Do another capillary blood test after 15 min and repeat resugaring if necessary.

For adults on a high-protein (and low-carbohydrate) diet:

- Resugaring with a high-protein food supplying lactose: for example, 200 ml of milk for 60 kg.
- Protein powder can be added if needed

A personalised document (or prescription) is provided to the patient and his/her family with an emergency card once the disease has been discovered.

#### Cardiological care

##### Cardiac failure treatments

In case of symptomatic diastolic cardiac failure, low doses of loop diuretics (minimum effective dose) can provide significant functional improvements.

Angiotensin-converting-enzyme inhibitors can be initiated, in the presence of myocardial fibrosis and/or systolic dysfunction, although their benefits remain hypothetical.

Betablockers could also provide functional benefit, in the presence of signs of diastolic or systolic cardiac failure and/or a left intraventricular obstruction with a maximum gradient over 50 mmHg [4].

Bradycardic calcium channel blockers can be proposed, as a second-line treatment, if beta-blockers are contra-indicated or badly tolerated, but associating both treatments is not generally necessary.

In the exceptional cases of atrial fibrillation, the treatment is the same as for general population with effective anticoagulant therapy. In the same way, the very rare cases of progression to systolic cardiac failure should receive a conventional treatment of cardiac failure [4].

##### Preventing sudden death

In cardiomyopathies, sudden death prevention relies on installing implantable defibrillators in patients at high risk of cardiac events. This is notably the case in the primary prevention of hypertrophic cardiomyopathies of sarcomeric origin. It does not appear appropriate to use the same treatment algorithms in hypertrophic cardiomyopathies associated with GSD III, since the level of rhythm risk appears lower. It is, however, difficult to estimate this precisely because of the low prevalence of this pathology and the absence of large-scale series with sufficiently long monitoring times. A few rare cases of sudden death have been reported in both children and adults (from 4 months to 36 years) for which the rhythm mechanism was unfortunately not recorded [4, 16, 26]. No cases of sustained ventricular tachycardia or high-grade conduction abnormalities have been reported. Given these elements, the indication of an implantable defibrillator, installed subcutaneously as a first-line treatment because of the absence of conduction abnormalities, should be discussed on a case-by-case basis for patients with the most severe hypertrophic cardiomyopathies, especially when this is associated with ventricular hyperexcitability and the presence of myocardial fibrosis on MRI [4].

##### Ketone bodies

The use of synthesised ketone bodies (sodium beta hydroxybutyrate) in several daily intakes (between 400 and 900 mg/kg/day in children, in 4 to 5 intakes per day) can also limit or even improve cardiac damage. Like for low-carbohydrate and high-protein diet, the underlying hypothesis is to provide the cardiac muscle with another energy substrate, easier to use than glycogen. The prescription is limited to specialised units in rare situations [67].

#### Muscular care

The care provision for muscles depends on the musculoskeletal work-up [38–41].

##### Physiotherapy

Depending on muscular work-up, each specific deficit should be worked on. Particular attention should be paid to Achilles tendon retractions, frequent in these patients. Static stretching of the triceps surae could be done, alongside balance exercises to gently work by proprioception on ankle muscular weakness. Distal motor skill exercises in the upper limbs can prove useful, given the frequency of writing difficulties. In cases of misalignment of the spinal column (lordosis, kyphosis), postural rehabilitation should be planned [31, 32].

Aerobic exercises can be proposed depending on the patient’s strength and endurance, during consultations but also as regular adapted physical activity (after cardiological work-up), in order to stimulate the fatty acid oxidation metabolic pathway.

Transfer training can be useful for both mobile and non-mobile patients.

For patients with little or no mobility, hydrotherapy—if available—can prove beneficial, as can joint mobilisations and muscle stretching. It is essential to ensure non-mobile patients are raised.

##### Occupational therapy

Depending on functional deficit assessment, technical aids could improve problems linked to fine motor skills damaged by distal weakness. Adaptive equipment could help with cutting, writing, using a keyboard or opening jars for example.

Driving adaptations can be proposed when both strength and grip function of upper limbs are compromised, or if lower limb deficit requires using hand controls.

In cases of loss of mobility, the correct positioning of the wheelchair should be checked.

##### Physical medicine and rehabilitation (PM&R)

Orthopaedic insoles can be prescribed in cases of alignment alteration (joint hypermobility, increased width of the base of support, anteverted pelvis, knee valgum and recurvatum, hindfoot valgus and/or forefoot varus). Leg lifters can be considered for the more severe forms, as well as for distal instabilities and weaknesses of the lower limbs in adults. A PM&R physician can also be approached to coordinate the rehabilitation of these complex patients.

#### Care provision for hepatic complications

For many years, the only care required is clinical, biochemical and radiological monitoring.

There can be discussions on whether to carry out a biopsy of a hepatic nodule that is growing rapidly and/or whose appearance is changing on imaging.

A liver transplantation can also be discussed, when the disease has evolved to cirrhosis, or even hepatocellular carcinoma. While awaiting a liver transplant, conventional cirrhosis management is essential [69, 70].

Alcohol consumption is prohibited since it can put patients in situations of risk of hypoglycaemia and worsen hepatic lesions.

#### The role of liver transplantation

Liver transplantation corrects the liver disease and no publications have reported a recurrence of the disease on the graft. However, few publications have described liver transplants in GSD III patients. In adults, the main indication for transplant was cirrhogenic evolution [69, 70]. In the livers removed from these patients, hepatocellular carcinoma lesions were detected. Three paediatric patients also received a liver graft from a living donor. The main indication, in these three children, was poor quality metabolic control [70]. They were operated on between the ages of 2 and 6 years. None had cardiomyopathy. We should also mention a 39-year-old patient who received a triple transplant, liver/heart/kidney because of the coexistence of cirrhosis, severe cardiomyopathy with pulmonary arterial hypertension and kidney failure.

The post-operative data show correction of metabolic perturbations and improvement in the general condition. However, the transplant does not correct any potential peripheral myopathy nor any potential cardiomyopathy [69, 70]. Very few GSD III patients have received a liver transplant, and it appears that there is an indication to maintain the high-protein diet to continue treating the muscular impairment.

To date, liver transplantation is a last resort procedure. It should be considered when there is cirrhosis, especially if episodes of decompensation and/or liver dysfunction have occurred.

Hepatocellular carcinoma may also be considered an indication in some cases.

#### Therapeutic patient education (TPE)

Therapeutic education is a set of activities making it possible to:

- Provide awareness-raising, information and teaching centred on the patient and his/her family.
- Understand the disease and its treatments
- Allow the patient to participate in the care and take control of his/her health condition.
- Provide psychological and social support to help the patient (and his/her family)

Therapeutic education is initially given to parents, then progressively to the patient using tools adapted to his/her age and level of understanding. Particular attention should be paid to adolescents who may become less compliant, as for all patients living with a chronic disease [71].

The aims of the TPE sessions are to assess the patient’s dietary knowledge (so as to propose adapted sessions), and ascertain whether he/she has acquired autonomy in terms of both treatment and disease.

Information should cover the following elements [71]:

- The progression of the disease from childhood to adulthood.
- The fact that the disease is genetic, inherited and definitive, and requires lifelong monitoring (or surveillance).
- The “patho-physiological” mechanisms: enzyme deficiency, the organs affected and the consequences on the metabolism.
- The main risks during childhood: hypoglycaemia and failure to thrive
- Self-tests: functioning of the blood glucose meter, capillary blood tests at home, realising and analysing blood glucose cycles at home.
- The resugaring protocol in case of hypoglycaemia
- The main risks in adulthood: progression of the liver disease and muscular impairment
- What to do when pregnant
- The emergency card
- The treatments

As concerns diet, TPE aims at achieving:

- An adaptation and division of food depending on the lifestyle and physical activities of the patient.
- A good knowledge of nutrients and food groups
- A correct choice of food to meet the objectives of the dietary treatment depending on age and cardiac damage:
  o The benefit and need for large amounts of food rich in complex carbohydrates (equivalences) during childhood, and the use of raw and cooked starch.
  p How to prepare raw starches: Maïzena® and Glycosade®
  q Training to prepare the mixture for night feeding (know how to make a ‘home-made’ mixture while following hygiene rules)
  r The benefit of and need to increase the intake of proteins (equivalences between the high-protein foods with good biological value), and addition of high-protein dietary complements: protein powders
  s Introduce a high-protein, low-carbohydrate and normal-calorie diet depending on any potential cardiac damage and its severity
  t Adapt the diet to life situations (pregnancy, etc.) and age
  u Avoid excessive weight gain
- Master the practice of nocturnal enteral feeding at home:
  o Positioning the tube and checking its position, if nasogastric tube
  p Knowing how to change the gastrostomy button
  q Knowing how to program the nutrition pump and connect it up.

In practice, it is best to proceed by small steps to obtain compliance to a specific diet:

- Learning to “GSD III eat”:
  o Giving practical keys to deal with constraints of the specific diet (equivalences, menu ideas, recipes, etc.) and learn to eat “as balanced as possible” to build a normal social life that is essential in the long term (adaptations for festive events, meal baskets, etc.)
  p Forming good habits: no added sugar, no sweets, not too many cakes, etc.
- Helping the patient to identify high-protein foods that he/she likes
- Help the patient to gradually decrease intake of bread and raw starches
- Introduce a specific diet (high-protein, low-carbohydrate and normal-calorie) depending on any potential cardiac damage and its severity

TPE also aims at helping with integration at school through a *Projet D’Accueil Individualisé* (Individualised Reception Plan) and provide dental advice such as toothbrushing techniques. It should be noted that eating disorders can make it difficult or even impossible to instigate the diet.

#### Child/adult transition

The American College of Medical Genetics recommendations for caring for GSDIII, published in 2010, contain no specific recommendations on the transition from childhood to adulthood. Filière G2M (national network for metabolic diseases)’s general recommendations concerning the child/adult transition of patients with inherited metabolic diseases can be applied to people with GSD III [71]. In practice, the transition is a long process which should lead to the patient being transferred to a unit specifically for adult patients, when available. Ideally, the concept of this transfer should be explained to patients and their families around 12 years of age. The first consultations without the parents can then be gradually proposed. The paediatric team should draft a transition plan detailing the medical/dietary and social elements needed for the transfer. Mixed consultations (paediatric team/ adult medicine team) should be organised at the paediatric unit and continue in the adult structure. The transfer can then take place if the clinical situation has been stable for the duration of one year (good metabolic balance, absence of rapidly evolutive complications, absence of major problems with compliance, etc.). Following this, the consultations in the adult units are initially held in the presence of parents, then without them, if possible (the aim being to support the patient to start managing their condition independently). Joint paediatric–adult review meetings, joint therapeutic eduction programmes and regular collaborations should be encouraged [71].

#### Medical–social care

Like for all chronic diseases, every consultation should be an opportunity to reassess different adaptations and support that patients can benefit from:

- 100% reimbursement of medical costs by the national social security system, under long-term health condition n° 17 (metabolic disease).
- Producing a medical certificate for the MDPH (*Maison Départementale des Personnes Handicapées/*French departmental disability service) to access financial compensation for multidisciplinary care package. For adults, the muscular impairment can be such that it becomes necessary to obtain the status of disabled worker (RQTH: *Reconnaissance de la Qualité de Travailleur Handicapé*/Recognition of the Status of Disabled Worker). Additionally, the patient can have the right to a Disabled Adult Benefit (*Allocation Adulte Handicapé* / AAH) and a car sticker to access disabled parking facilities. These different types of support should be explained to patients who are eligible, and a social worker can be assigned to help them obtain these rights.
- Recourse to a healthcare provider to instal and support home enteral feeding.

In paediatrics, the referring doctors of all schoolchildren should draft an Individualised Reception Plan *(Projet D’Accueil Individualisé.PAI).* This “PAI” is destinated to the teaching team at school, and describes what the patient is allowed to do, what he must avoid, and what to do in case of emergency. Parents can also be eligible for a daily 'Parental Presence' Allowance (*Allocation Journalière de Présence Parentale* /AJPP). This is a financial compensation that may be given, should one of them be obliged to stop working to take care of her/his affected child.

#### Emergency care

In GSD III, all situations involving fasting for longer than normal or poor dietary intake carry a risk of severe hypoglycaemia: enforced fasting (surgery, invasive procedure, childbirth, etc.), vomiting, diarrhoea, fever with low appetite, etc. In these situations, patients should be able to monitor their capillary blood glucose levels regularly and an emergency protocol should be started without delay.

The basic principle during these circumstances is to provide a continuous flow of glucose that is sufficient to maintain normal blood glucose levels, i.e. > 0.5 g/L (or 2.8 mmol/L). The flow rate depends on age (see table below).

**Table:** 

Age	0–24 months	2–4 years	4–14 years	> 14 years—adult	MAX FLOW RATE
Infusion flow rate	6 ml/kg/h (8-10 mg/kg/min)	5 ml/kg/h (6 mg/kg/min)	3.5 ml/kg/h (4 mg/kg/min)	2.5 ml/kg/h (2 mg/kg/min)	120 ml/h

Glucose infusion rate as a function to the age of the patient

Most often, glucose intake is provided by a 10% polyionic glucose infusion in a hospital. In some specific cases, it could also be provided by continuous enteral feeding via a nasogastric tube or gastrostomy, if the patient is already following a dietary regime comprising enteral nutrition, and if there are neither food intolerances nor diarrhoea [1–3]. The mixture and glucose infusion rate to be administered are generally the same as those supplied during the night. This solution can be set up at home, depending on the patient’s level of understanding, provided that blood glucose levels are carefully monitored.

In all cases, patients should be driven rapidly to the closest emergency department, since any risky situation should trigger the emergency protocol, and increase the frequency of blood glucose monitoring. Like in most metabolic diseases, patients with GSDIII should carry an emergency card and an emergency certificate explaining the protocol in detail. The certificate should be understandable to any non-specialised physician involved in the emergency care.

#### Therapeutic prospects

There are some new therapeutic prospects emerging for the medium term. These are based on work carried out in animal models (historically a dog and more recently a mouse) obtained using techniques involving the invalidation of the gene coding for the debranching enzyme.

Of particular interest are the prospects of gene therapy using recombinant viral vectors, to attempt to correct, at least partially, the enzyme deficiency in both liver and muscles [72].

The first results obtained in animals for muscular impairment are encouraging. The studies must be continued before a clinical trial in humans can be considered.

### Particular situations

#### Pregnancy/contraception

Contraception for women should be rigorously discussed. Indeed, the risk of developing adenomas means particular attention should be paid to oestrogen treatments. It is easier to use progestogen alone. However, the risk of osteopenia associated with this type of treatment should be considered.

Women with GSD III have a higher risk of polycystic ovary syndrome without necessarily reducing their fertility [49].

The pregnancy should be subject to closer monitoring:

- There is a risk of cardiac decompensation linked to both physiological haemodynamic modifications and to stopping the angiotensin-converting-enzyme inhibitors because of their teratogenic effect. This justifies a cardiac assessment before planning a pregnancy and a discussion about the estimated level of risk. Clinical and ultrasound cardiological monitoring should be planned, depending on the presence or absence of cardiomyopathy and its severity.
- Blood glucose monitoring should be more frequent since the increased sugar requirements linked to hormone modifications in pregnancy can lead to metabolic imbalance. It is often necessary to increase the number of meals and to adapt the supply of carbohydrates and proteins. It is sometimes necessary to restart nocturnal enteral feeding for a few weeks during the pregnancy. Additionally, it is important to prevent the formation of ketone bodies as much as possible, since these could induce premature contractions and present a risk for the foetus. Pregnancy-related nausea and vomiting should also be limited as much as possible since this could reduce food intake and result in hypoglycaemic episodes.

Pre-pregnancy and post-natal monitoring should be undertaken to screen for the appearance or modification of any liver adenomas.

It also may be necessary to increase the physiotherapy.

Birth should be planned, if possible in a specialised centre. Serum glucose infusion should be offered during labour and until normal food intake is re-established. A caesarean section may be needed if myopathy is severe. There are no particular precautions to take apart from the glucose serum drip (see emergency care) if emergency anaesthesia is required for a caesarean section.

In all cases, every pregnancy should be planned and monitored in a centre of reference specialised in GSD III care.

#### Vaccinations

All mandatory vaccinations in the vaccination calendar are advised and there are no contra-indications for the other recommended vaccinations. However, patients and their families, children and adults, should be encouraged to receive the flu vaccine every winter, since this viral infection carries an increased risk of decompensation.

Additionally, because of the risk of cirrhosis in these patients, hepatitis A and B vaccinations are strongly recommended. Equally, pneumococcal vaccine is recommended in patients presenting significant cardiopathy.

#### Travel

Given the risk of potentially severe hypoglycaemic attacks in cases of extended fasting, and the existing differences among health systems abroad, every trip to a foreign country should be carefully planned.

The patient should ensure he/she has several copies of the emergency certificate in his/her possession, in French but also translated into the language of the country in question, or at least into English.

Blood glucose meter and all supplies needed (test strips, finger pricker lancets, finger prickers) should be carried in hand luggage (to reduce the impact of potentially losing baggage). This also goes for G30% vials, used when rapid resugaring is required, any enteral feeding equipment (in cases of a dietary regime involving night feeding), and the dietary products needed (including Maïzena® or Glycosade®). Ideally, adequate quantities to cover the entire trip should be packed, since these products may not be available in foreign countries. It should be noted that Maïzena® can be found in most countries and can easily replace Glycosade® for occasional use. It is best to ask the patient’s referring physician to write out a certificate that quickly explains the disease and the need to carry this equipment in cabin luggage.

It is preferable to make contact with the closest hospital to the holiday accommodation before leaving, should an emergency hospitalisation be necessary.

In cases of doubt as to the ability of the hospital in the holiday destination to provide emergency care, all measures should be taken to enable an emergency repatriation to Europe or a developed country: repatriation insurance, return plane ticket that can be modified free of charge, etc. If emergency treatment conditions do not appear to be available, the trip is discouraged.

All precautions needed to avoid at-risk circumstances (notably acute diarrhoea or intercurrent infection) should be repeated to the patient before leaving: only drink bottled or disinfected/filtered water, wash foods before eating, avoid raw foods and privilege well-cooked foods, avoid ice-cubes, etc.

Ideally, both planning and organisation for the trip should be discussed in advance, in a dedicated consultation**.** When the trip is in Europe, it is best to procure a European social security certificate to facilitate the administration and avoid having to pay healthcare fees.

#### Pathologies and other intercurrent treatments/anaesthesia

All intercurrent pathologies resulting in poor food intake or a change in the normal diet put the patient at risk of severe hypoglycaemia, and should be avoided. Additionally, in all situations of unusual fasting, the emergency protocol should be applied without delay, with the introduction of continuous glucose supply [1–3].

##### Surgery and anaesthesia

Any type of surgery (involving pre-operative fasting) requires an infusion of 10% polyionic glucose solution following the patient’s emergency protocol, to be introduced from the beginning of the fasting period, with regular blood glucose monitoring (every 2–4 h) to adapt the infusion flow rate in order to maintain blood glucose levels between 0.60 g/L (3.3 mmol/L) and 1.2 g/L (6.6 mmol/L). This intravenous supply should not be stopped until the patient is able to completely restart his/her normal diet orally.

To avoid any errors resulting in hypoglycaemia, and all risks of inhalation, these patients should be under strict fasting and infused the day before the procedure (in general at least 8 to 10 h of fasting), especially if they are taking Glycosade®.

Non-depolarising anaesthetic agents should be avoided because of the muscular impairment. Indeed, succinylcholine is not recommended because of the associated risk of rhabdomyolysis.

In cases of chronic hepatic damage such as cirrhosis, pre-operative coagulation tests are mandatory.

The surgical risk of a potential cardiomyopathy should always be discussed between the referring cardiologist or cardio-paediatrician and the anaesthetist, especially for high-risk surgeries.

##### Other treatments

All medication that could lead to hypoglycaemia should be avoided or used with caution if it proves to be indispensable (for example, beta-blockers to treat hypertrophic cardiomyopathy). In the days following the introduction of these drugs, blood glucose levels should be monitored more closely. If type 2 diabetes occurs in these patients, anti-diabetic treatment should be prescribed after seeking the opinion of a specialist [51].

Long-term corticosteroids, growth hormone and oestrogens are contra-indicated because of their interference with carbohydrate metabolism and their propensity to trigger the development of hepatic adenomas or rhabdomyolyses. Statins should be avoided, but their prescription can be discussed on a case-by-case basis, depending on the patient’s cardio-vascular risk.

Finally, all medicinal prescriptions should be reassessed and adapted in cases of proven cirrhosis [46].

#### Sport

Patients with GSD III legitimately question whether they can practise sport since some types of exercise are badly tolerated. Additionally, the risk of hypoglycaemia should not be ignored, given the pathological accumulation of hepatic glycogen and the increased consumption of glucose by skeletal muscles during physical activity. The cardiac risk should also be considered and cardiology examination should be performed before starting physical training.

However, not doing any physical exercise is not advisable. Indeed, a sedentary lifestyle is not only responsible for skeletal muscular atrophy or cardiac maladaptation, but it also causes metabolic alterations such as the preferential use of glycogen (defective in GSD III) and the reduction of the oxidative capacity of fatty acids, which constitutes an alternative functional pathway in this pathology.

Unfortunately, there is a lack of publications on regular exercise in GSD III which prevents us from providing patients with precise recommendations on practising sport. Currently, only the following elements from the scientific literature can be used to answer this question:

- Given that the myophosphorylase is intact in GSD III, a small quantity of glucose can be liberated at the start of the physical effort, which probably explains the absence of muscle spasms upon effort, of myoglobinuria and of recurrent rhabdomyolysis.
- With our current state of knowledge, low-intensity physical exercise (< 70% VO2 max) appears beneficial for patients living with GSD III [29]. However, resistance exercises and high-intensity sport (≥ 60% of maximum strength) should be practised with caution, or even not at all. Additionally, violent sports should be avoided in the presence of hepatomegaly, as should activities favouring joint distension in patients presenting hyperlaxity linked to muscular weakness.
- In all cases, physical activity should be adapted to the patient’s potential.
- Finally, it has been demonstrated that an intravenous glucose injection improves tolerance to exercise in patients living with GSDIII [29]. We can therefore assume that oral ingestion of glucose would also improve tolerance to effort and limit the risk of hypoglycaemia. However, this remains to be proven and the modalities of this intake are yet to be established (dosage, how long before the effort). In practice, at this stage of knowledge, we can only suggest a snack before exercise, made of milk and raw starch [29].

#### Schooling

The school should be informed of the risk of hypoglycaemia, of the specific diet and the need for corn starch supplements and/or protein powders. A PAI should be established and updated every time the diet is changed.

Additionally, myopathy and cardiopathy should be taken into account, if needed, to authorise or adapt school sports activities, so that the pupil is not disadvantaged compared to classmates.

In general, schooling for children with GSD III proceeds normally. However, for some children with concentration or learning difficulties it may be necessary to provide special assistance such as AESH (*Accompagnement d’Elèves en Situation de Handicap/*Support for Disabled Pupils) or reduce their school hours.

School trips are not contra-indicated. Parents and teachers should discuss these in advance.

A brochure aimed at teaching teams explaining GSD has been produced by a work group from Filière G2M (national network for metabolic diseases). (Link: http://www.tousalecole.fr/sites/default/files/medias/integrascol/documents/Accueillir%20à%20l%27école%20un%20enfant%20avec%20une%20glycogénose%20hépatique.pdf).

### Clinical and paraclinical monitoring of patients

All patients should be periodically monitored in a reference centre in order to guarantee a high-quality assessment and clinical follow-up.

Clinical follow-up examination should be identical to that performed at the initial assessment. The frequency of consultations is adapted to clinical evolution. Some complementary tests should be carried out systematically.

Additionally, self-monitoring at home should be prescribed, either by installing a continuous glucose monitoring [68], or by the patient carrying out capillary blood tests (or by parents for children). This monitoring is particularly important at every significant change in lifestyle in adulthood (new working hours or mealtimes, new physical activity, etc.) and during childhood.

#### Objectives

- Obtain precisions about the evolution (progression or regression of a previously known impairment, identification of a previously unknown impairment).
- Screen for the appearance of a comorbidity.
- Adapt the dietary treatment in function to the change in the patient’s lifestyle, or his/her growth, compliance or fasting tolerance.
- Assess psychological, family and socio-professional consequences of the disease.
- Refer the patient to other professionals according to his/her needs.
- Inform the patient of the ongoing clinical and therapeutic research protocols.
- Assess the patient’s knowledge about his/her disease.
- Answer the patient’s questions.
- Take the patient’s life plans into account (travel, procreation, etc.).
- Organise the transition to adult medicine.

### Frequency and content of the consultations

**Table:** 

Monitoring parameter		Frequency
	Childhood–adolescence	**Adulthood**
Clinical monitoring		
Clinical examination*	3 times a year for the first 3 years then every 6 months	Every 6 to 12 months
Dietary interview	3 times a year for the first 3 years then every 6 months	Every 6 to 12 months
Muscle function assessment: Neurologist Physio / occupational therapist / PM&R	At diagnosis if possible Then 1x/yr	1x/yr
Medical–social assessment	At diagnosis then on request	On request
Neuropsychological tests	Depending on clinical context, before decisions concerning schooling	Depending on clinical context
Paraclinical monitoring		
Biochemical work-up **	3 times a year for the first 3 years then every 6 months	Every 6 to 12 months
Abdominal ultrasound	Every 12 months	Every 6 to 12 months
Hepatic MRI	Annually after the age of 10	Annually
Osteodensitometry	Every 5 years after the age of 10	Every 5 years
ECG	Annually	Annually
Holter monitor	Every 1–2 years, if MHC or symptoms suggesting rhythm disorder	Every 1 to 2 years, if MHC or rhythm symptoms
Echocardiography	Every 6–12 months	Annually

*Details of the clinical examination:

Growth in height and weight in children; weight, height and BMI in adults

Size of liver and signs of cirrhosis

Complete muscular examination (+ fatigability)

Dyspnoea and palpitations, cardiac examination

Dental assessment

Assessment of psychological condition

**Details of biochemical work-up:

“Routine” work-up performed at each consultation:

Pre- and post-prandial blood glucose-lactate cycle

Complete hepatic work-up: ASAT, ALAT, GGT, ALP, total and conjugated bilirubin

CPK

Lipid profile: cholesterol, triglycerides

Urea, creatinine

Ionogram

Complete Blood Count

PT, factor V

Uric acid

Annual work-up, to be added at least once a year to the routine biochemical work-up:

Alpha-foetoprotein

BNP or pro-BNP

25-OH-Vitamin D

Vitamins B1, B6, B12

Adjusted serum calcium, serum phosphorus, serum magnesium

Iron work-up: iron, ferritin, transferrin saturation coefficient

HbA1c especially in adults

This table is indicative but both clinical context and appearance of complications can evidently lead to increase the frequency of patient monitoring

## Data Availability

Not applicable.

## Abbreviations

AESH: Accompagnement d’Elèves en situation de handicap/support for disabled pupils
AGL: Amylo-alpha-1,6-glucosidase, 4 alphaglucanotransferase
AJPP: Allocation Journalière de Présence parentale/daily 'Parental Presence' allowance
ALAT: Alanine aminotransferase
ALP: Alkaline phosphatase
ANSES: Agence nationale de sécurité sanitaire de l’Alimentation, de l’environnement et du travail (national agency for sanitary safety of food, environment and work)
ARA: Arachidonic acid
ASAT: Aspartate aminotransferase
BMI: Body mass index
BNP: Brain natriuretic peptide
CEF: Continuous enteral feeding
CPDPN: Centre pluridisciplinaire de diagnostic prénatal/multidisciplinary centre for prenatal diagnosis
CPK: Creatine phosphokinase
DHA: Docosahexaenoic acid
ECG: Electrocardiogram
ENMG: Electroneuromyogram
GGT: Gamma glutamyl transferase
GSD: Glycogen storage disease
GSD III: Glycogen storage disease type 3
HAS: Haute autorité de santé/French National Authority for Health
HbA1c: Glycated haemoglobin
LDH: Lactate dehydrogenase
MDPH: Maison départementale des personnes handicapées/French departmental disability service
MFE: Meat–fish–egg
MFM (Scale): Motor function measure scale
mg/kg/min: Milligramme per kilogramme per minute
MRI: Magnetic resonance imaging
NASH: Non-alcoholic steatohepatitis
NGS: New generation sequencing
NRP: Nutritional reference for the population
NT pro BNP: N-terminal pro-brain natriuretic peptide
PAI: Projet D’accueil individualisé/individualised reception plan
PAS: Periodic acid–schiff
PM&R: Physical medicine and rehabilitation
PNDS: Protocole national de diagnostic et de soins/French national protocol for diagnosis and healthcare
TEI: Daily total energy intake
TPE: Therapeutic patient education
